# Protein surface charge of trypsinogen changes its activation pattern

**DOI:** 10.1186/s12896-014-0109-5

**Published:** 2014-12-28

**Authors:** Karin Buettner, Thomas Kreisig, Norbert Sträter, Thole Zuchner

**Affiliations:** Institute of Bioanalytical Chemistry, Center for Biotechnology and Biomedicine, Universität Leipzig, 04103 Leipzig, Germany; Current address: Octapharma Biopharmaceuticals GmbH, Im Neuenheimer Feld 590, Heidelberg, 69120 Germany

**Keywords:** Protein design, Protein expression, Protein-interaction, Protein engineering, trypsin

## Abstract

**Background:**

Trypsinogen is the inactive precursor of trypsin, a serine protease that cleaves proteins and peptides after arginine and lysine residues. In this study, human trypsinogen was used as a model protein to study the influence of electrostatic forces on protein–protein interactions. Trypsinogen is active only after its eight-amino-acid-long activation peptide has been cleaved off by another protease, enteropeptidase. Trypsinogen can also be autoactivated without the involvement of enteropeptidase. This autoactivation process can occur if a trypsinogen molecule is activated by another trypsin molecule and therefore is based on a protein–protein interaction.

**Results:**

Based on a rational protein design based on autoactivation-defective guinea pig trypsinogen, several amino acid residues, all located far away from the active site, were changed to modify the surface charge of human trypsinogen. The influence of the surface charge on the activation pattern of trypsinogen was investigated. The autoactivation properties of mutant trypsinogen were characterized in comparison to the recombinant wild-type enzyme. Surface-charged trypsinogen showed practically no autoactivation compared to the wild-type but could still be activated by enteropeptidase to the fully active trypsin. The kinetic parameters of surface-charged trypsinogen were comparable to the recombinant wild-type enzyme.

**Conclusion:**

The variant with a modified surface charge compared to the wild-type enzyme showed a complete different activation pattern. Our study provides an example how directed modification of the protein surface charge can be utilized for the regulation of functional protein–protein interactions, as shown here for human trypsinogen.

**Electronic supplementary material:**

The online version of this article (doi:10.1186/s12896-014-0109-5) contains supplementary material, which is available to authorized users.

## Background

Protein–protein interactions are based on non-covalent interactions of amino acids on protein surfaces via van der Waals forces, hydrogen bridges, electrostatic interactions and hydrophobic effects. Other processes that can be influenced by electrostatic interactions include for example protein folding and protein stability, protein denaturation and solubilization or precipitation of proteins [1]. In addition, the binding of substrates by enzymes, the subsequent catalytic processes and also the formation of protein–protein complexes can be based on electrostatic interactions.

The human enzyme trypsinogen shows a distinct distribution of protein surface charges and may be an attractive model protein to study the influence of electrostatic forces on protein–protein interactions. The activation of trypsinogen by its natural activation enzyme, the serine protease enteropeptidase, is determined by a close interaction between the two proteases. Trypsinogen is the inactive precursor of trypsin (PRSS1, human cationic trypsinogen), which can cleave proteins and peptides after lysine and arginine residues and is activated by the membrane-bound enteropeptidase via cleavage of the N-terminal activation peptide located on the trypsinogen surface. The resulting trypsin itself activates other pancreatic zymogens of the digestive pathway [2]. Besides the specific activation of trypsinogen by enteropeptidase it is well known that trypsinogen can show autoactivation [3].

This trypsinogen autoactivation process is mostly based on the fact that active trypsin can activate trypsinogen by cleaving off its activation peptide in a similar way as occurs with the activation enzyme enteropeptidase. Subsequently, the newly formed trypsin can then accelerate the activation of other trypsinogen molecules in a cascade reaction.

Several mutations that influence the autoactivation of human trypsinogen have been published and are discussed in the context of the development of pancreatitis [4,5]. A number of studies have investigated the influence of different mutations on the autoactivation process [6-8]. In these studies, autoactivation was initiated by addition of a small amount of trypsin. As the same amount and type of trypsin was used throughout these studies, this ensures that autoactivation was characterized in a comparable way.

Some studies [3,9] have also hypothesized that trypsinogen autoactivation may not require the presence of either active trypsin or active enteropeptidase, but that trypsinogen itself could have an inherent, though minimal, catalytic function and could therefore activate itself. However, physiological conditions of course include the possibility that trypsinogen activation could also occur to a minor extent through the non-specific cleavage by proteases other than trypsin or enteropeptidase. In addition, it may be experimentally challenging to exclude the presence of even the smallest amount of active protease in order to investigate this problem. We therefore decided to study the autoactivation process by adding a small defined amount of active trypsin to ensure comparability with published studies and congruent results.

**Figure 1:**
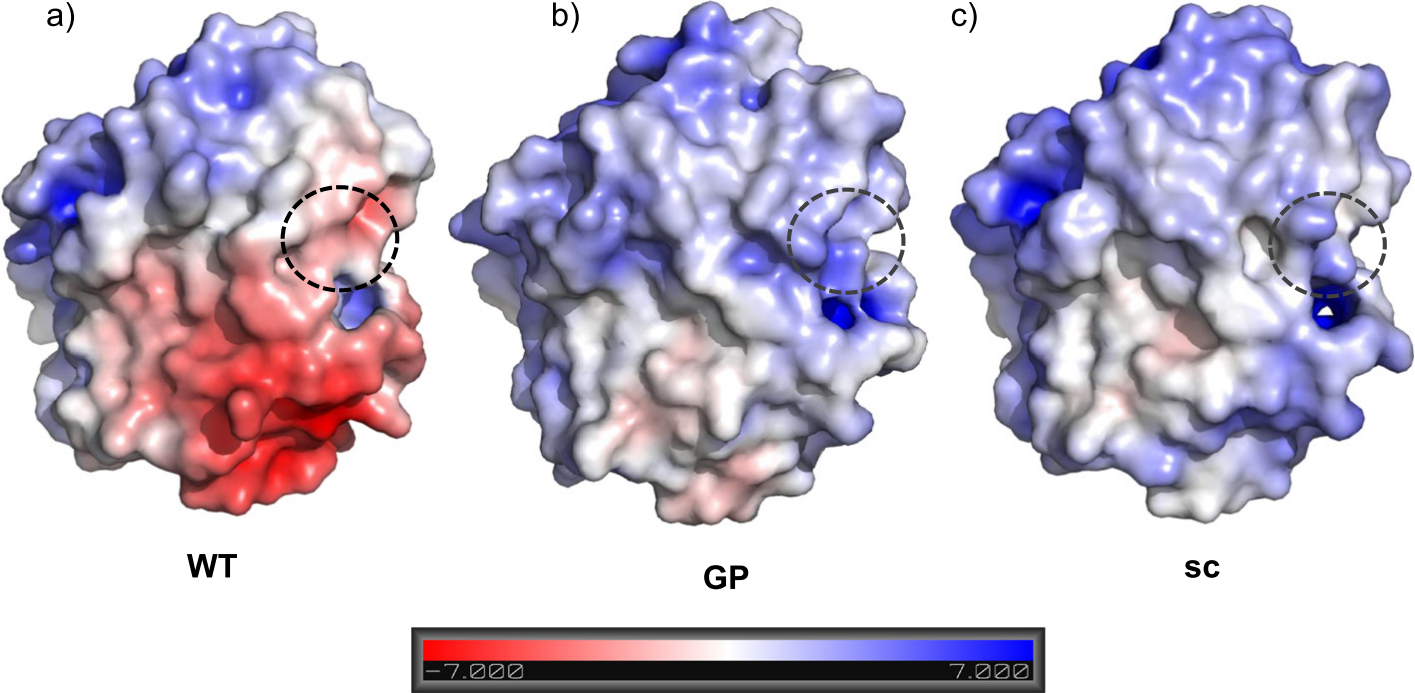
**Electrostatic surface potential of (a) human cationic trypsinogen (“WT”) in comparison to (b) modeled guinea pig trypsinogen (“GP”) and (c) modified human trypsinogen (“sc”).** The range of electrostatic surface potential is shown from -7 kT/e (red color) to +7 kT/e (blue color). N-terminal amino acids (V25 based on structure of trypsinogen 1TGN) are marked with a black circle. The substrate-binding cleft is located on the rear side of the shown model structures.

Trypsinogen autoactivation as observed for the human enzyme differs from that observed in other organisms [10]. For example, trypsinogen from guinea pig is defective in autoactivation under conditions where human cationic and anionic trypsinogen rapidly autoactivate [8]. The reason for this different autoactivation behavior has not been clear until now. The guinea pig variant has a sequence identity of 75% with human cationic trypsinogen. However, when we compared the number of charged amino acids between human and guinea pig trypsinogen, we found a striking difference in the number of charged amino acids (26 vs. 42 in human cationic trypsinogen). More than this, when we looked at the position of these charged amino acids within the protein model, it turned out that a striking difference in the surface charge distribution between human and guinea pig wild-type trypsinogen can be observed (Figure 1a and b). Based on this observation, our aim was to study the influence of a modified protein surface charge of human trypsinogen on specific enzyme properties, and in particular on the autoactivation. Our hypothesis was, that the distinct distribution of surface charges could potentially influence the activation pattern of the enzyme. To study this in detail, we also had to consider that guinea pig trypsinogen contains a lysine at position 79 instead of the typical glutamine residue found in other mammalian trypsinogens. The functional role of this side chain is unclear, but the Glu79Lys mutation (E79K) in human cationic trypsinogen seems to be associated with chronic pancreatitis and also leads to a lower autoactivation compared to the wild type [11].

The rational optimization of surface charge–charge interactions has been described before, for example as a strategy to enhance protein stability [12]. The advantages of this method are that mutations on the protein surface are less likely to significantly affect the core structure and therefore the function of the protein [13]. One example is the generation of highly thermoresistant antibodies by increasing their surface charge [14]. The mutation of surface residues to charged amino acids is also known to decrease protein aggregation. Previously, the method of supercharging was demonstrated to increase the resistance of green fluorescent protein (GFP) to temperature-induced aggregation [15,16] to increase the solubility of a human enteropeptidase by more than 100-fold [17]. These studies reported the influence of protein surface charge on rather general protein properties such as solubility, aggregation properties and thermoresistance.

In this paper, we describe the modification of human trypsinogen by the exchange of several amino acid residues on the protein surface and its effect on specific properties of the protein such as the protein–protein interaction behavior of trypsinogen with enteropeptidase and active trypsin.

## Results

### Rational protein design

**Figure 2:**
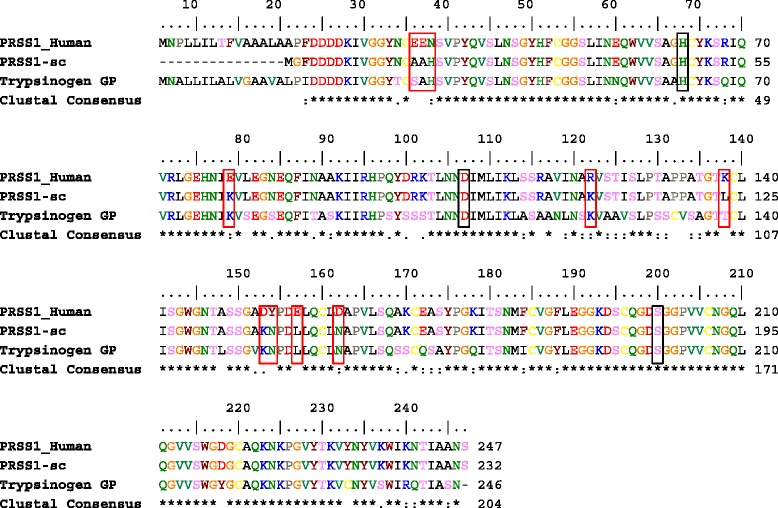
**Sequence alignment of human trypsinogen [GenBank: NP_002760] with surface-charged mutant (PRSS1-sc) and guinea pig trypsinogen (Trypsinogen GP, [GenBank: NP_001166359]).** Mutated positions of PRSS1 are E31A, E32A, N33H, E79K, R122K, K138L, D153K, Y154N, E157L, and D162N. The catalytic triad of trypsinogen is marked with black boxes. Exchanged amino acids in the mutant are indicated in red boxes. ClustalW [http://embnet.vital-it.ch/software/ClustalW.html] was used for the sequence alignment. Alignment was edited by Bioedit [http://www.mbio.ncsu.edu/bioedit/bioedit.html].

In this work, we examined the influence of the protein surface charge on protein–protein interactions of the serine protease human trypsinogen. Trypsinogen is an interesting model protein because it can be activated both by trypsin and by enteropeptidase. The resulting active trypsin can then contribute to the further activation of other trypsinogen molecules. As a result of the increasing amounts of active trypsin, a cascade reaction of autoactivation is observed. One interesting trypsinogen variant that shows nearly no autoactivation is the trypsinogen from guinea pig [8]. After we had done a sequence comparison between guinea pig and human cationic trypsinogen, several residues could be identified that are different in charge (Figure 2).

Interestingly, when we looked at the spatial distribution of these charged amino acids in the 3D structure of the protein, these charged amino acids are mostly located on the enzyme surface and distributed in a very distinct way (Figure 1). Inspired by this finding, we created a human trypsinogen mutant in which we tried to include a similar surface charge distribution as found by us for the guinea pig version. Although this kind of influence of a proteins surface charge on its activation pattern has never been described before, we aimed to examine its influence on the activation pattern of human trypsinogen.

After the amino acid sequence alignment of both variants, ten residues were identified that could lead to a different protein surface potential for trypsinogen. To prevent the undesirable loss of enzyme activity or specificity by mutations in the generated human trypsinogen variant, it was necessary to engineer the protein in such a way as to minimize the influence on the active center of the enzyme. Thus, mutated positions were checked to be on the surface of the protein and, in particular, to be distant from the activation sequence of the enzyme (distance >10 Å). The mutations were then performed in a way that the predicted electrostatic potential of the variant matched that of guinea pig trypsinogen.

In detail, the following ten mutations were introduced: E31A, E32A, N33H, E79K, R122K, K138L, D153K, Y154N, E157L, and D162N. We decided not to mutate position 138 like in the sequence of guinea pig trypsinogen, which has a threonine at this position to prevent posttranslational modification of OH-groups. The same consideration applied to position 31 and 154 [18,19].

In total, the trypsinogen sc variant has 16 negatively charged residues compared to the 22 in the wild-type enzyme and 21 positively charged residues compared to the 20 in the wild type. Thus, the net charge was increased from −2 to +5. Several mutations are known to potentially influence the autoactivation and autolysis of human trypsinogen like E79K and R122H [11,20]. Furthermore, the position R122 has the potential to influence the autolysis of the protein. It was therefore necessary to examine the influence of these positions on the human trypsinogen so as to allow a clear conclusion about the influence of the protein surface charge as compared to the effect of these point mutations. The effect of these mutations will be discussed later in this study.

### Autoactivation of trypsinogen at pH 8

**Figure 3:**
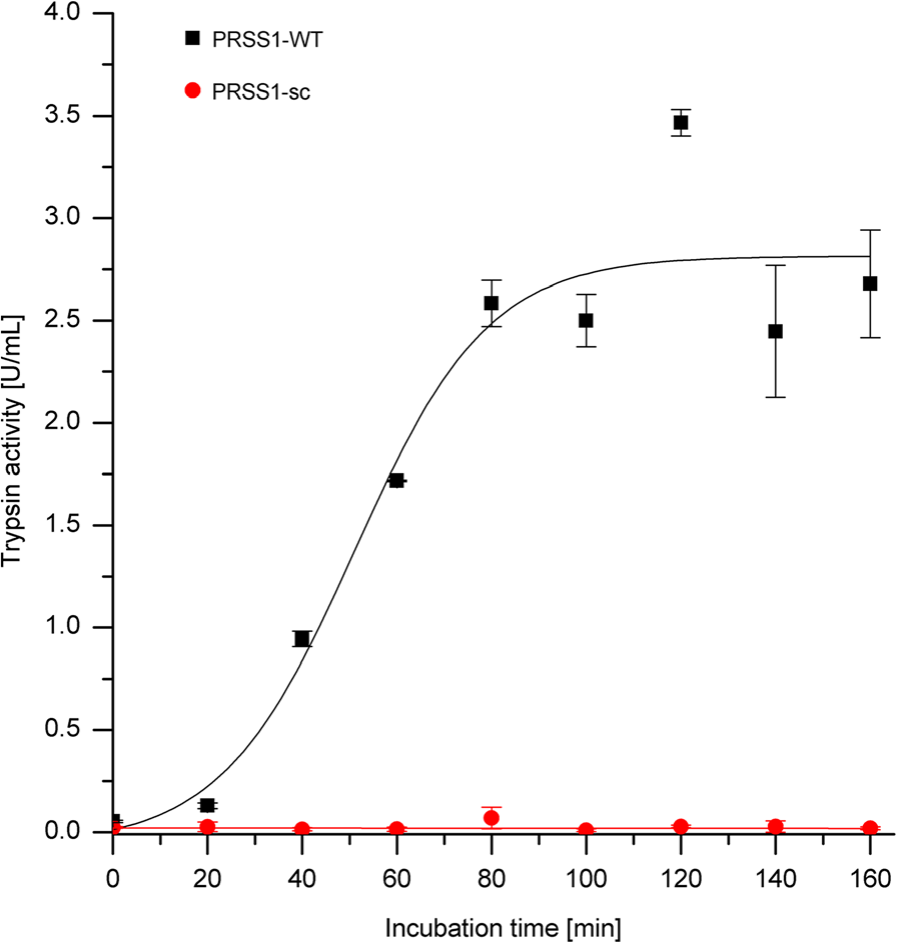
**Autoactivation of trypsinogen at pH 8.** Trypsinogen autoactivation was measured in 100 mmol/L Tris-HCl pH 8, 1 mmol/L CaCl_2_ at 37°C with 2 μmol/L trypsinogen and 10 nmol/L trypsin as an initial starting concentration. Trypsin activity was determined with Cbz-GPR-pNA as substrate.

The autoactivation of human wild-type trypsinogen and the surface-charged variant (PRSS1-sc) was investigated at pH 8 and 37°C for several hours. To initiate the autoactivation and in accordance with several other studies [6-8], 10 nmol/L trypsin was added to 2 μmol/L trypsinogen. The time-course of the autoactivation was quantified by determination of the trypsin activity (Figure 3).

**Figure 4:**
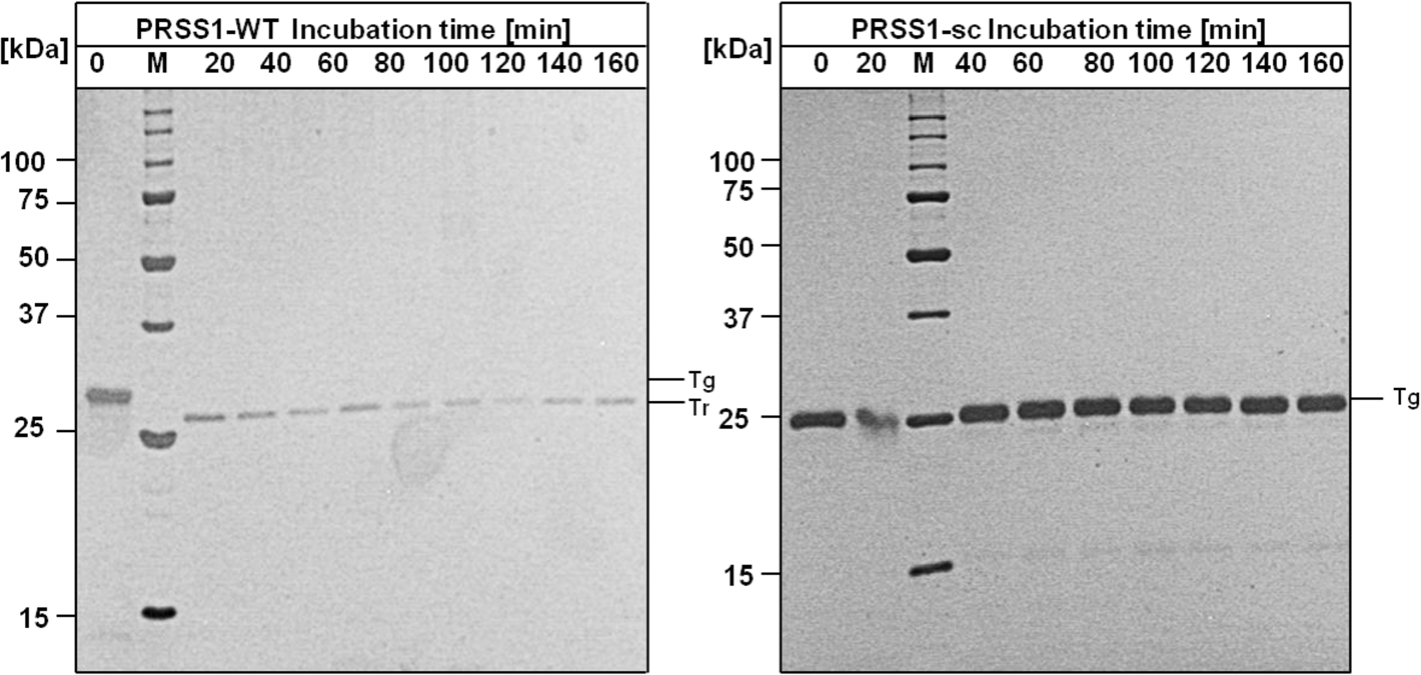
**SDS-PAGE analysis of autoactivation of human trypsinogen.** The samples were analyzed on a 15% reducing SDS-PAGE gel using silver staining. Reactions were terminated by adding SDS-PAGE sample buffer followed by an immediate denaturation step for 5 min at 95°C (Tg = trypsinogen; Tr = trypsin).

In agreement with previous studies, the human wild-type enzyme showed a high autoactivation [7]. This high autoactivation rate results in the complete activation of trypsinogen within 80 min. PRSS1-WT exhibits an activation rate constant of 30.9 nmol/L/min for activation of trypsinogen by trypsin. In contrast to this, the surface-charged PRSS1-sc variant showed nearly no detectable activity during the whole incubation time under the same conditions. This means, that the surface-charged trypsinogen variant shows a dramatically reduced activation rate when using trypsin as the activating enzyme. An activation rate constant of 0.00545 nmol/L/min, which is more than 5000 times slower than for the wild-type enzyme, was determined for the PRSS1-sc mutant. These results were also confirmed by SDS-PAGE analysis (Figure 4).

In SDS-PAGE, the band corresponding to human wild-type trypsinogen is detectable only in the 20 and 40 min samples. Even after 40 min the lower molecular weight band corresponding to the active trypsin molecule is the dominant species. In contrast, the surface-charged trypsinogen showed no detectable shift in the molecular weight and also no degradation of the enzyme. This confirms the finding of the activity measurement (Figure 3), i.e., that nearly no autoactivation occurs in the surface-charged variant.

**Figure 5:**
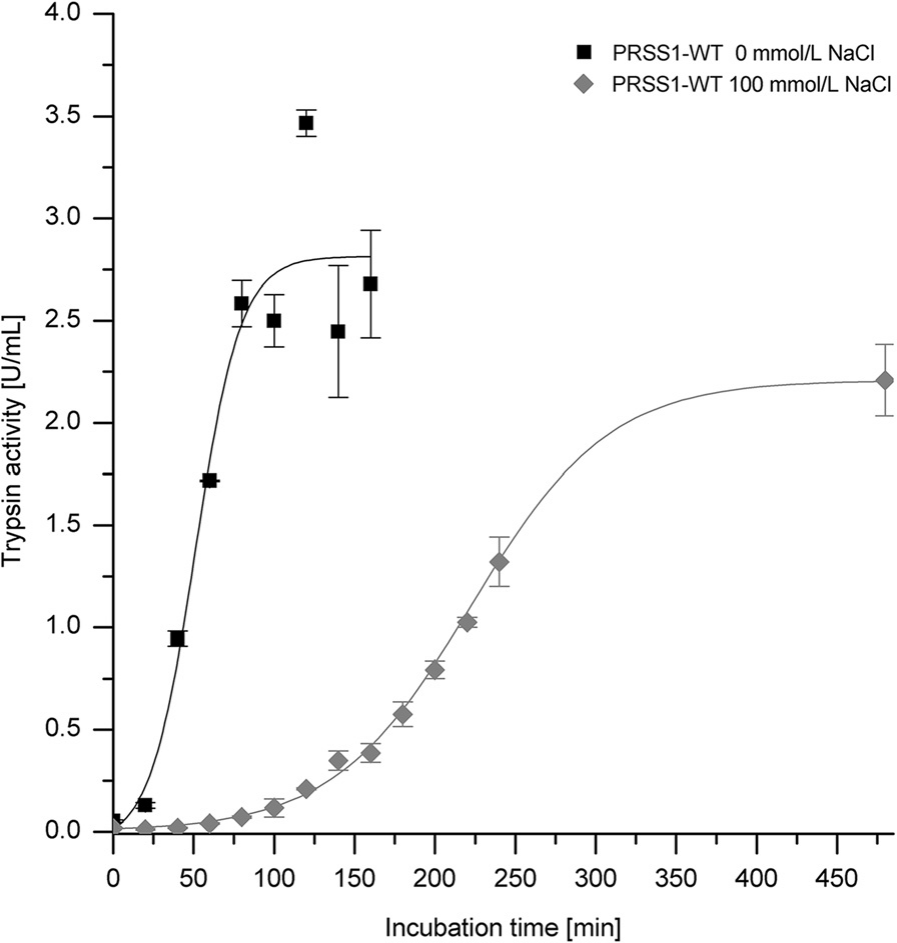
**Autoactivation of trypsinogen at pH 8 and different salt concentrations.** Trypsinogen autoactivation was measured in 100 mmol/L Tris-HCl pH 8, 1 mmol/L CaCl_2_ and 100 mmol/L NaCl at 37°C with 2 μmol/L trypsinogen and 10 nmol/L trypsin as an initial starting concentration. Trypsin activity was determined with Cbz-GPR-pNA as substrate.

The autoactivation of wild-type and surface-charged trypsinogen was also investigated at higher ionic strength to further confirm our hypothesis that autoactivation is influenced by the surface charge of the trypsinogen molecule and therefore the binding between the molecules. We could observe a decreased autoactivation of the wild-type trypsinogen compared to the experiment without the addition of salt (Figure 5).

The PRSS1-sc mutant contains the mutation E79K, besides the other mutations described above. The E79K mutation has previously been described to cause a decreased autoactivation [11]. Therefore, we also generated the point mutant PRSS1-E79K and investigated its autoactivation properties in the same way as for wild-type trypsinogen. Under the conditions used in this study, the E79K mutant showed an autoactivation that was comparable to that of the wild-type trypsinogen (Additional file 1: Figure S1). This finding seemingly contradicts the previously published result [11] where this mutant did not show autoactivation. However, in the study published by Teich et al., no trypsin was added to initiate the autoactivation and the activation behavior was not measured for a prolonged time. We additionally determin the autoactivation behavior of wild-type trypsinogen and PRSS1-E79K with the addition of a lower amount of trypsin (1 nmol/L). We could confirm that autoactivation mechanism is influenced by the amount of added trypsin. The addition of a lower amount of trypsin led to a decreased autoactivation for wild-type trypsinogen and also for mutant PRSS1-E79K (Additional file 1: Figure S1). We can therefore conclude that the E79K mutation did not significantly contribute to the observed decreased autoactivation in PRSS1-sc under the conditions used in this study.

### Activation with enteropeptidase

**Figure 6:**
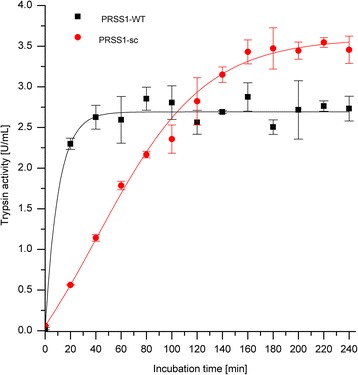
**Activation of trypsinogen with human enteropeptidase.** Enteropeptidase-mediated trypsinogen activation was measured in 0.1 mol/L Tris-HCl pH 8, 1 mmol/L CaCl_2_ at 37°C with 2 μmol/L trypsinogen and hEPl-Sc-C112S (1 nmol/L final concentration).

In its physiological setting, trypsinogen is activated by enteropeptidase. We therefore examined the activation behavior of the human wild-type and the surface-charged trypsinogen by human enteropeptidase to see if the surface-charged variant could be activated in a normal manner. The characterization of the trypsin activity after enteropeptidase activation showed that the surface-charged trypsinogen could be activated to normal wild-type levels after 100 to 120 min (Figure 6). In comparison, the wild-type trypsinogen was already completely active after 40 min. The activity of surface charged trypsinogen increases up to 3.5 U/mL compared to wild-type trypsinogen with 2.5 U/mL activity. These differences are based on different specific activities of the variants (PRSS1-sc 69 U/mg and PRSS1-WT 54.6 U/mg). The detailed kinetic parameters of wild-type and surface charged trypsinogen can be found in Table 1. The surface-charged and wild-type trypsinogen were incubated with enteropeptidase over 4 hours at 37°C without showing a decreasing activity, indicating that both trypsinogen mutants are in a stable conformation.

**Table 1:** **Kinetic parameters for trypsinogen with substrate Cbz-GPR-pNA**

	***K***_**m**_ **[μmol/L]**	***k***_**cat**_ **[1/s]**	***k***_**cat**_ **/** ***K***_**m**_ **[L/μmol/s]**
PRSS1-WT	78.0 ± 12.2	324.1 ± 13.8	4.7 ± 0.5
PRSS1-sc	129.8 ± 8.4	489.0 ± 18.9	3.7 ± 0.3
PRSS1-WT*	27 ± 3	99 ± 5	3.7

*Parameters as given by [31].

The activation rate constant for the activation of PRSS1-WT by enteropeptidase was 86.4 nmol/L/min, which is slightly higher (a factor of 6.5) compared to the activation rate constant for PRSS1-sc (13.3 nmol/L/min). This means that the activation rate of mutant PRSS1-sc is slowed down by a factor of more than 5000 when trypsin is used as the activating enzyme, whereas the same mutant can still be activated to normal levels when enteropeptidase is used as the activating enzyme, albeit at a 6.5-fold slower rate.

**Figure 7:**
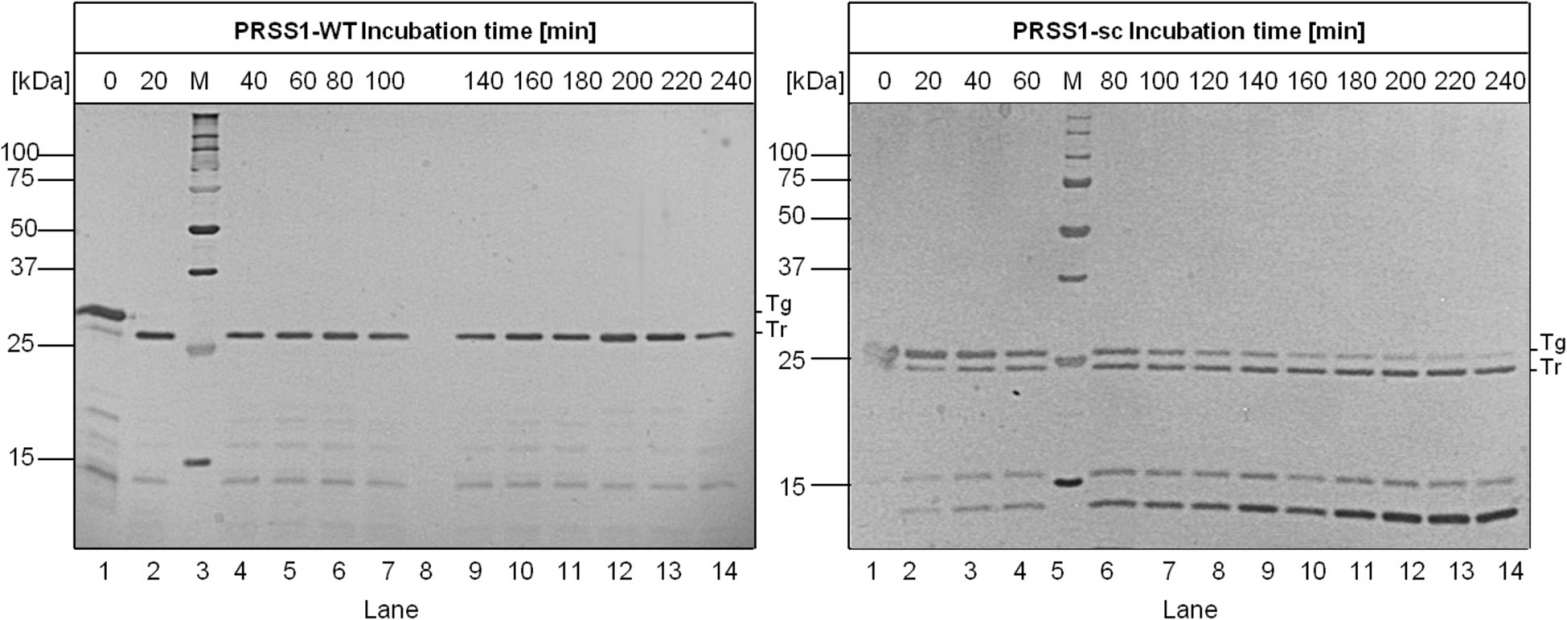
**Activation of trypsinogen with human enteropeptidase.** The samples were analyzed on a 15% reducing SDS-PAGE gel using silver staining. Reactions were terminated by adding SDS-PAGE sample buffer followed by an immediate denaturation step for 5 min at 95°C.

The course of this human enteropeptidase–mediated activation behavior of the surface-charged trypsinogen vs. wild-type trypsinogen was also examined via SDS-PAGE analysis (Figure 7). The results showed that it is possible to activate the surface-charged mutant to a full extent by using human enteropeptidase.

Two degradation bands are visible after activation. In previous studies, it was shown that these degradation bands are connected to the cleavage of the Arg122–Val123 peptide bond [21]. Arg122 is exchanged to a lysine residue in the surface-charged trypsinogen, which indicates that the Lys122–Val123 bond can also be cleaved by trypsin.

### Kinetic parameters

To determine whether the mutations of surface-charged trypsinogen had an influence on the enzyme activity, Michaelis–Menten kinetic parameters were determined. Although the *K*_m_ value of the surface-charged trypsinogen was slightly increased compared to wild-type trypsinogen, no major differences for *K*_m_, *k*_cat_ or *k*_cat_/*K*_m_ values were observed (Table 1, Additional file 2 and Additional file 3). PRSS1-WT had a specific activity of 54.6 U/mg. In comparison, PRSS1-sc had a slightly increased specific activity of 69 U/mg. The catalytic efficiency (*k*_cat_/*K*_m_ value) showed no difference for wild-type trypsinogen and surface-charged trypsinogen, indicating that the active center of the enzyme was not influenced.

To summarize these results, we found that the directed mutation of the trypsinogen surface charge led to an almost complete loss of trypsinogen autoactivation, while the enzyme could still be completely activated by its natural activation enzyme human enteropeptidase. These results were confirmed by activity measurements and by SDS-PAGE. Although the kinetic parameters of wild-type and surface-charged human trypsinogen for small peptide substrates were essentially the same, the activation of the surface-charged variant by human enteropeptidase occurred slightly slower than in the case of the wild-type enzyme.

## Discussion

Enzyme autoactivation is a specific type of protein–protein interaction that is not only known to trypsinogen, but also for a number of other enzymes, including proteases such as pepsinogen and prothrombin [3,22-24]. Although different autoactivation mechanisms for the above mentioned enzymes exist, the intermolecular mechanism behind the trypsinogen autoactivation could potentially be a model for investigating the influence of protein surface charges on protein-protein interactions.

In this study, a human trypsinogen mutant was created with a distinct distribution of a surface charge pattern. This idea was based on our finding, that a trypsinogen variant from guinea pig which is defective in autoactivation shows a very specific distribution of surface charges. When we created a human variant in which a similar surface charge distribution was introduced, this led to a mutant of the human trypsinogen that showed a dramatically reduced autoactivation.

Lawrence et al. demonstrated that proteins can be highly supercharged [15] without abolishing their folding or function. Specifically, the net charge of GFP was increased by several amino acid exchanges that led to variants which remained entirely soluble upon thermal and chemical treatment. Supercharging altered the intermolecular properties of the proteins, providing aggregation resistance and the ability to associate in folded form with oppositely charged macromolecules [15]. The supercharging of GFP was quite intense, with an increase of the net charge from −7 to +36 or −30 [15,16]. In the structure-based design of supercharged, highly thermoresistant antibodies, the best mutant had an increased positive charge by eight [14]. In that study, fewer substitutions in the single-chain variable fragment antibody were required to achieve improvements in thermostability and affinity. The method of surface charging was also used by us in an earlier study to improve the solubility and thermostability of human enteropeptidase [17]. In this case, several mutations were introduced that led to six additional negative charges. The increased net charge led to a more than 100-fold higher solubility of the enzyme.

In this study, the surface charge of trypsinogen was changed by mutation of several amino acids, which resulted in a variant with practically no autoactivation.

Numerous amino acid substitutions have been discussed in the literature with respect to their effect on the stability of human trypsin. A mutation that was described in the literature and also examined in this study is located at position Arg122. This position is known to influence the autolytic – not the autoactivation – properties of the enzyme. The autolytic cleavage of the Arg122–Val123 peptide bond was suggested to trigger rapid trypsin degradation by increasing structural flexibility and exposing further tryptic sites [25]. Mutations at position 122, such as exchange to histidine or cysteine, inhibited the autolysis of trypsin [26-28] and enhanced the autoactivation of trypsinogen [26,28]. In this study, the mutation R122K did influence the self-cleavage of the enzyme, as shown by the SDS-PAGE gels. Furthermore, no decreasing activity was determined during activation with enteropeptideas and no increasing effect on the autoactivation was observed. We assume that the effect of a mutation at position 122 is too weak to cause a significant effect in the context of the surface charging we performed.

In this study, we could also show that the surface charge influences the autoactivation behavior of trypsinogen by investigating this property at different salt concentrations. Based on our results we could conclude that autoactivation is influenced by the surface charge of the protein because the autoactivation is decreased at higher ionic strength conditions.

The net charge of the modified protein was changed from a negative charge (−2) in the wild-type trypsinogen to a positive charge (+5) in the mutant. In total the human surface-charged trypsinogen has 16 negatively charged residues and 21 positively charged residues. More specifically, human wild-type trypsinogen shows a distinct negative surface potential on one side of the protein (Figure 1). Interestingly, these negative charges are mostly present close to the activation sequence of the protein. We therefore hypothesize that the negatively charged domains in the human wild-type trypsinogen allow an interaction with the positively charged surface areas of another trypsinogen molecule, and that this interaction leads to the autoactivation of the enzyme. While this study does not prove that the wild-type autoactivation mechanism is directly controlled by the surface charge of the protein and combinatorial effects may apply, it is nevertheless clear that a modified surface charge strongly affects the autoactivation behavior. At the same time, enteropeptidase can still activate the modified trypsinogen with an only moderately reduced efficiency, indicating that the interaction of these two proteins is determined by a different pattern of electrostatic interactions.

Additionally, it is also possible that reduced autoactivation of surface-charged trypsinogen is based on other effects like post-translational modifications, higher stability of the enzyme and/or conformational changes. As already stated, the advantage of a rational protein design is that the mutations on protein surface are less likely to significantly affect the core structure and therefore the function of the protein [13]. Trypsinogen mutant was designed by reducing possibility of post-translational modifications and we could also show that the catalytic efficiency has not changed. We also could show that autoactivation of wild-type trypsinogen is decreased at higher ionic strength (Figure 5). This proves that the modified surface charge of the protein can not only control protein–protein interactions in general, but also selectively, as in case of our model enzyme trypsinogen. This selective influence of a protein surface charge on its interaction with other proteins has not been demonstrated by us or, to the best of our knowledge, by anybody else until now.

## Conclusion

In this study, we were able to reduce the autoactivation of human trypsinogen by the modification of its protein surface charge. The mutation of ten amino acids led to an increase in the net charge of the enzyme and resulted in a reduced autoactivation, which is probably due to specifically disturbed protein–protein interactions between two trypsin(ogen) molecules. The activation of trypsinogen by enteropeptidase was, in contrast, only influenced in a minor way. This gives a strong indication, that the protein surface charge of trypsinogen has a specific regulatory function on the protein-protein interaction pattern of the enzyme.

## Methods

### Materials

Ecotin was expressed in *E. coli* BL21 as described by Pál et al., 1996 [29,30] and purified via a trypsin affinity column. Purified ecotin was coupled to a pre-packed NHS-activated Sepharose column (GE Healthcare) according to the manufacturer’s protocol.

Host strains *E. coli* DH5α, *E. coli* BL21 (DE3) and plasmid pET28a(+) were from Novagen (Darmstadt, Germany). *N*-Cbz-Gly-Pro-Arg-*p*-nitroaniline was purchased from Bachem AG (Bubendorf, Switzerland). Human enteropeptidase hEPL-Sc-C112S was prepared according to Simeonov et al., 2010 [17]. Chemicals were of the highest purity available.

### Construct human trypsinogen mutant

Full-length cDNA of PRSS1 (human cationic trypsinogen) was from Deutsches Ressourcenzentrum für Genomforschung GmbH (Berlin, Germany). The plasmid (PRSS1 N-2X in vector full ORF shuttle clone IOH 43660) contained the full-length cDNA of PRSS1 [GenBank: NM_002769.4] and a stop codon.

The gene was amplified via polymerase chain reaction (PCR) using the forward primer 5'-ggagatataccatgggctttgatgatgatgacaag-3' and the reverse primer 5'-cttgtcatcatcatcaaagcccatggtatatctcc-3' resulting in the N-terminal end of the protein Met-Gly-Phe-Asp_4_-Lys. It was shown that the modification of the N-terminus of trypsinogen enhances the expression level [31]. For the reason in the present study the N-terminus was also changed to Met-Gly-Phe-Asp_4_-Lys.

The PCR was performed by heating for 2 min at 95°C, followed by 18 cycles of 95°C for 30 s, 54°C for 1 min, 68°C for 1 min and an additional extension time at 68°C for 2 min. The amplified PCR product was analyzed via a 1% agarose gel and purified via a gel extraction kit (Qiagen, Hilden, Germany). The purified DNA sequence was cloned into the pET28a vector via restriction sites *Nco*I and *Xho*I. *E. coli* BL21 (DE3) was transformed with construct pET28a-PRSS1dAP.

The DNA of the surface-charged trypsinogen was obtained via gene synthesis (Eurofins MWG Operon, Ebersberg, Germany). The N-terminal end was modified as described above for wild-type trypsinogen.

### Expression and purification of human trypsinogen

Trypsinogen was expressed as inclusion bodies in *E. coli* cells. *E. coli* BL21 (DE3) cells harboring the expression vector with the PRSS1 gene were grown in TB medium with 30 μg/mL kanamycin at 37°C until an OD_600_ of 0.8 to 1 was reached. The expression was induced by adding 1 mmol/L IPTG and glucose to a final concentration of 1% (w/v). The cells were incubated for 18 h at 25°C and then harvested by centrifugation (20 min, 5000 *g*).

Inclusion bodies were purified by resuspending the cell pellet in 100 mmol/L Tris-HCl (pH 7.0) 1 mmol/L EDTA and 3 mmol/L MgCl_2_ containing 0.1 mg/mL lysozyme and 10 μg/mL DNase I. The cells were disrupted with a high-pressure homogenizer (two passages of 1000 bar). After incubation for 30 min at 24°C, disrupted cells were centrifuged (20 000 *g*, 4°C, 30 min).

The inclusion bodies comprising the trypsinogen were prepared from the insoluble fraction after the cell disruption as follows: inclusion bodies were resuspended in IB washing buffer I (20 mmol/L EDTA pH 8.0, 500 mmol/L NaCl, 2% Triton X-100), stirred for 30 min at room temperature and centrifuged for 30 min at 20 000 *g*. The resulting pellet was washed again with IB washing buffer I and twice with IB washing buffer II (20 mmol/L EDTA, 100 mmol/L Tris-HCl, pH 7.0).

1 g of inclusion bodies were solubilized in 20 mL 4 mol/L Gnd-HCl, 0.1 mol/L Tris-HCl pH 8.0, 5 mmol/L EDTA, and then reduced with DTT (final concentration 30 mmol/L) for 20 min at 60°C. Afterwards, DTT was removed via a desalting column (GE Healthcare). The refolding was done via fast dilution of protein (final concentration 100 μg/mL) in 0.7 mol/L Arg-HCl (pH 8.6), 1 mmol/L EDTA, 2 mmol/L reduced glutathione and 2 mmol/L oxidized glutathione. The refolding mixture was incubated for 16 h at 4°C. The refolded trypsinogen was subsequently purified via an ecotin affinity column.

Protein concentrations of trypsinogen were determined spectrophotometrically at 280 nm using an extinction coefficient of 37525 L/mol/cm for PRSS1-WT [calculated by Protparam from http://www.expasy.org/].

### Trypsin activity assay

Trypsin activity was determined using the synthetic substrate *N*-Cbz-Gly-Pro-Arg-*p*-nitroaniline (final concentration 200 μmol/L) with an absorption readout at 405 nm in 0.1 mol/L Tris-HCl (pH 8.0) 1 mmol/L CaCl_2_ at 25°C.

### Activation of trypsinogen by enteropeptidase

Trypsinogen (2 μmol/L final concentration) was activated by human enteropeptidase hEPl-Sc-C112S (1 nmol/L final concentration) in 0.1 mol/L Tris-HCl (pH 8.0) 1 mmol/L CaCl_2_ at 37°C. At given times, aliquots for the trypsin activity assay and SDS-PAGE analysis were taken.

The activation rate constant was calculated for wild-type and surface charged trypsinogen based on the linear slope of the increasing trypsin activity. Together with the specific activity we then calculated how many trypsinogen molecules are activated by trypsin per minute.

### Autoactivation of trypsinogen

Trypsinogen in 50 mmol/L HCl was diluted to a 2 μmol/L final concentration in 0.1 mol/L Tris-HCl pH 8.0 in the presence of 1 mmol/L CaCl_2_. Autoactivation was initiated by addition of 10 nmol/L bovine trypsin and the reaction mixture was incubated at 37°C. At given times, aliquots for the trypsin activity assay and SDS-PAGE analysis were taken.

### SDS-PAGE

Autoactivation of trypsinogen was also visualized by gel electrophoresis and silver staining. Samples were heat denatured at 95°C for 5 min and loaded onto 15% mini-gels (Miniprotean; Bio-Rad Laboratories, Hercules, CA, USA). Silver staining was performed according to Shevchenko et al., 1996 [32].

### Modeling

Models for the structure of guinea pig trypsinogen and human trypsinogen were generated with the program MODELLER using the bovine trypsinogen structure [PDB: 1TGN] as template [33]. The atomic coordinates of the structures were obtained from the Protein Databank at the RCSB [http://www.rcsb.org/pdb/home/home.do]. The model with the lowest DOPE score [34] obtained from MODELLER was refined with fragment-guided MD simulation (FG-MD) [http://zhanglab.ccmb.med.umich.edu/FG-MD/] [35].

The program Coot was used for modeling of mutations in the human trypsin structure [36]. For visualization of the protein structure and the surface potential PyMol was used [37]. The surface potential was calculated by PDB2PQR [38,39].

## Additional files

Additional file 1:
**Autoactivation of wild-type and E79K trypsinogen at pH 8.** Trypsinogen autoactivation was measured in 100 mmol/L Tris-HCl pH 8, 1 mmol/L CaCl_2_ at 37°C with 2 μmol/L trypsinogen and 1 nmol/L or 10 nmol/L trypsin as an initial starting concentration. Trypsin activity was determined with CBZ-GPR-pNA as substrate. Additional file 2:
**Kinetic analysis of PRSS1-WT.** Kinetic parameters are given in Table 1. Enzyme was activated by human enteropeptidase (hEPl-Sc-C112S) for 200 min at 37°C in 100 mmol/L Tris-HCl pH 8.0, 1 mmol/L CaCl_2_. Assays were performed in 100 mmol/L Tris-HCl pH 8.0, 1 mmol/L CaCl_2_ and 10 μmol/L to 1000 μmol/ L CBZ-GPR-pNA at room temperature. The reaction was started by adding PRSS1 variants (12.5 ng) and monitored continuously for 5 min by an absorbance measurement at 405 nm (extinction coefficient for p-nitroaniline ε = 10092 L/mol/cm). Additional file 3:
**Kinetic analysis of PRSS1-sc.** Kinetic parameters are given in Table 1. Enzyme was activated by human enteropeptidase (hEPl-Sc-C112S) for 200 min at 37°C in 100 mmol/L Tris-HCl pH 8.0, 1 mmol/L CaCl_2_. Assays were performed in 100 mmol/L Tris-HCl pH 8.0, 1 mmol/L CaCl_2_ and 10 μmol/L to 1000 μmol/L CBZ-GPR-pNA at room temperature. The reaction was started by adding PRSS1 variants (12.5 ng) and monitored continuously for 5 min by an absorbance measurement at 405 nm (extinction coefficient for p-nitroaniline ε = 10092 L/mol/cm).
